# Assessing Vaccination Prioritization Strategies for COVID-19 in South Africa Based on Age-Specific Compartment Model

**DOI:** 10.3389/fpubh.2022.876551

**Published:** 2022-06-15

**Authors:** Chao Zuo, Zeyang Meng, Fenping Zhu, Yuzhi Zheng, Yuting Ling

**Affiliations:** School of Management Engineering and E-Commerce, Zhejiang Gongshang University, Hangzhou, China

**Keywords:** COVID-19, vaccination strategy, social contact, age structure, compartment model

## Abstract

The vaccines are considered to be important for the prevention and control of coronavirus disease 2019 (COVID-19). However, considering the limited vaccine supply within an extended period of time in many countries where COVID-19 vaccine booster shot are taken and new vaccines are developed to suppress the mutation of virus, designing an effective vaccination strategy is extremely important to reduce the number of deaths and infections. Then, the simulations were implemented to study the relative reduction in morbidity and mortality of vaccine allocation strategies by using the proposed model and actual South Africa's epidemiological data. Our results indicated that in light of South Africa's demographics, vaccinating older age groups (>60 years) largely reduced the cumulative deaths and the “0–20 first” strategy was the most effective way to reduce confirmed cases. In addition, “21–30 first” and “31–40 first” strategies have also had a positive effect. Partial vaccination resulted in lower numbers of infections and deaths under different control measures compared with full vaccination in low-income countries. In addition, we analyzed the sensitivity of daily testing volume and infection rate, which are critical to optimize vaccine allocation. However, comprehensive reduction in infections was mainly affected by the vaccine proportion of the target age group. An increase in the proportion of vaccines given priority to “0–20” groups always had a favorable effect, and the prioritizing vaccine allocation among the “60+” age group with 60% of the total amount of vaccine consistently resulted in the greatest reduction in deaths. Meanwhile, we observed a significant distinction in the effect of COVID-19 vaccine allocation policies under varying priority strategies on relative reductions in the effective reproduction number. Our results could help evaluate to control measures performance and the improvement of vaccine allocation strategy for COVID-19 epidemic.

## Introduction

The emergence of the novel coronavirus disease 2019 (COVID-19) has led to a global pandemic with serious implications for public health security. During this crisis, a large number of diagnostic protocols and treatment methods have been designed based on comprehension of the pathological characteristics of severe acute respiratory syndrome coronavirus 2 (SARS-CoV-2) (1, 2). The vaccines are considered to be important for the prevention and control of COVID-19, so many countries are developing COVID-19 vaccines based on the infection mechanisms of SARS-CoV-2 and its effect on host immunity (3, 4). However, many countries have experienced insufficient access to vaccines and the major vaccine manufacturers find it hard to ramp up production in a short time. In particular, the variants of the SARS-CoV-2 (Beta, Delta, and Omicron variant) have reduced the effectiveness of existing vaccines, which prompts some counties to take COVID-19 vaccine booster shots (three doses) and develop new vaccines to prevent substantial morbidity and mortality. The development of effective vaccination strategies is critical given the limited availability of vaccines over the long term. It is well-known that vaccines should be allocated first to high-risk groups such as first responders and immunocompromised populations. What's worth exploring is the vaccine distribution of other groups after vaccination of high-risk groups.

One of the characteristics of COVID-19 is that the susceptibility, infectivity, severity, and mortality of the disease vary by age (5–7). Studies indicated that the susceptibility to infection usually increases with age, however, younger adults, especially those under 35, tend to experience the highest cumulative infection rates (6). Meanwhile, older adults have a higher mortality compared to younger individuals, mortality for those aged under 65 years range from 0% to 42%; and for those aged above 65 years range from 0% to 56% (7). Vaccination priority given to different age groups will affect the cumulative morbidities and mortalities. Moreover, the rate of infection relies on the social contact patterns (represented by the contact matrix), which depicts the contact degree between age groups, and is the linear combination of the location-specific matrices of household, school, workplace, and other locations (8). The epidemic can spread through the social network, which depends on pandemic contact pattern about the extent individuals interact with each other, and thus the contact patterns can effectively guide public health authority identify individual at high risk of infection and where an outbreak can be effectively prevented (9). Many studies consistently recommended that prioritizing younger populations who usually possess a higher contact rate exerts a greater effect on reducing morbidities relative to prioritizing older age groups (10, 11). Besides, the implementation of control measures, such as social distancing, lockdowns, and confinement on travel can slow the spread of pandemics and reduce morbidities (12). Several studies indicated that reasonable control measures substantially reduced the effective reproduction number in various regions (13). For example, relaxing restrictions can be considered to give priority to those less vulnerable age-brackets, which is presented because disease spread and mortality are apparently affected by the age distribution of the population (14). Daily testing volume is the mainstays of case finding, including asymptomatic and symptomatic infections, by identifying more infected people and then taking clinical treatment contributed to prevent the onward infection of others (15). In the absence of COVID-19 vaccine or shortage of medical resources, the implementation of large-scale rapid testing is an effective measure to curb transmission and death, particularly with asymptomatic transmission accountable for 44% of infections, thus increased testing volume is critical to the infection rates reduction (16). Vaccine availability and rollout speed can reflect the approximate time to vaccinate the target population, which promotes the vaccine coverage by continuous distribution to suppress the transmission of epidemics (17). Considering breakthrough infections resulted from the emergence of new variants, and waning immunity from primary COVID-19 vaccines, booster shots are an effective option for the prevention against COVID-19, which urges the adoption of more vaccines and faster rollout speed (18).

Thus, an effective vaccination priority strategy requires an understanding of the complicated interaction between age structure and age-specific social contact patterns and is combined with various hypothetical scenarios, such as the control measures, vaccine availability, detection rate, and rollout speed of vaccine, which also affect the spread of the COVID-19 epidemic. Meanwhile, the compartmental model is a very general modeling technique used for describing the flow patterns between the compartments of a system, which is often applied to the mathematical modeling of infectious diseases. Motivated by the above considerations, we construct an age-specific compartment model to evaluate the optimal distribution of limited COVID-19 vaccine availability across different age groups under various potential vaccine characteristics and hypothetical scenarios.

One of the dominant factors of the fourth wave in South Africa was the emergence of the Omicron variant. The public quickly understood that the Omicron variant had enhanced the infection rate of the delta virus (19). Accordingly, when the Omicron variant reached South Africa in November 2021, the number of cases and hospitalizations increased significantly. As of 6 February 2022, 27.92% of South Africans received two doses of vaccine, 4.99% of South Africans received one dose of vaccine, and only 1% had received the booster shot, the cumulative number of infections in South Africa reached 3.6 million (20). Therefore, we examine the effects of our proposed model and use pandemic contact matrices and actual epidemiological data in South Africa (1 November 2021–31 January 2022) to quantify and evaluate the effect COVID-19 vaccine prioritization policies have on cumulative morbidities and mortalities.

The study is organized as follows. In section model, we construct the age-specific compartment model. In section results, the numerical simulations are performed to assess the vaccination strategy. Finally, discussions are put forward in section discussions.

## Model

### Mathematical Modeling

**Figure 1:**
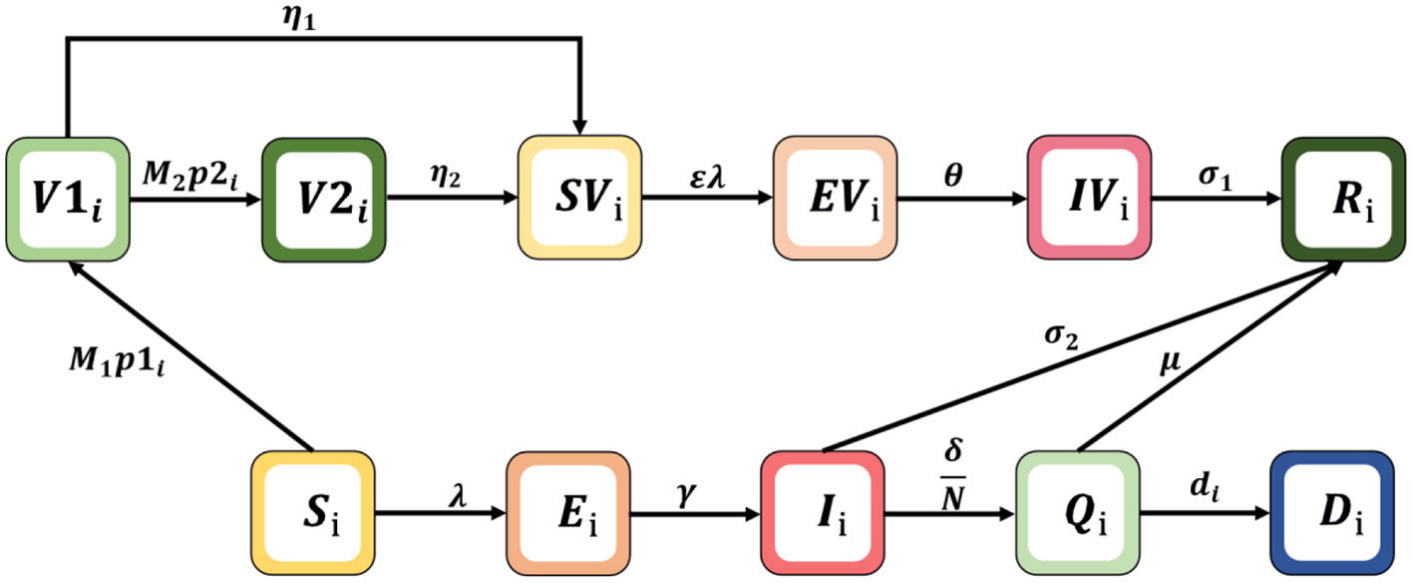
Schematic diagram of the mathematical model.

To simulate the transmission and vaccination process of COVID-19, an age-specific compartment model is constructed, and the population is divided into compartments according to the characteristics of each age group: *S*_*i*_= susceptible, *E*_*i*_= exposed, *R*_*i*_= recovered, *Q*_*i*_= hospitalized intensive care, *D*_*i*_= dead, *I*_*i*_= infected, *V*1_*i*_= vaccinated first doses, *V*2_*i*_= vaccinated second doses, *SV*_*i*_= susceptible and vaccinated, *EV*_*i*_= exposed and vaccinated, *and IV*_*i*_= infected and vaccinated. The age classes *i* = 1, 2, 3, 4, 5, and 6 represent individuals aged 0–20, 21–30, 31–40, 41–50, 51–60, and 60+ years, respectively. The schematic diagram of the model is shown in Figure 1.

The dynamic model is described by the following non-linear differential equation system:


(1) $$\{\begin{array}{c} \begin{array}{l} \frac{dS_{i}}{dt}=-S_{i}\;*\;\lambda_{i}-M_{1}\;*\;p1_{i}\\ \frac{dE_{i}}{dt}=S_{i}\;*\;\lambda_{i}-E_{i}\;*\;\gamma \;\\ \frac{dI_{i}}{dt}=E_{i}\;*\;\gamma -I_{i}\;*\;\frac{\delta }{N}-I_{i}\;*\;\sigma_{2}\;\\ \frac{dQ_{i}}{dt}=I_{i}\;*\;\frac{\delta }{N}-Q_{i}\;*\;\mu -Q_{i}\;*\;d_{i}\;\\ \frac{dD_{i}}{dt}=Q_{i}\;*\;d_{i}\;\frac{dR_{i}}{dt}=Q_{i}\;*\;\mu +I_{i}\;*\;\sigma_{2}+IV_{i}\;*\;\sigma_{1}\\ \;\frac{dV1_{i}}{dt}=M_{1}\;*\;p1_{i}-M_{2}\;*\;p2_{i}-V1_{i}\;*\;\eta_{1}\\ \frac{dV2_{i}}{dt}=M_{2}\;*\;p2_{i}-V2_{i}\;*\;\eta_{2}\\ \frac{dSV_{i}}{dt}=V1_{i}\;*\;\eta_{1}+V2_{i}\;*\;\eta_{2}\;-SV_{i}\;*\;\varepsilon \;*\;\lambda_{i}\;\\ \frac{dEV_{i}}{dt}=SV_{i}\;*\;\varepsilon \;*\;\lambda_{i}-\;EV_{i}\;*\;\theta \\ \;\frac{dIV_{i}}{dt}=EV_{i}\;*\;\theta -IV_{i}\;*\;\sigma_{1}\; \end{array} \end{array}$$

Where λ_*i*_ is the infection force for each age group, $\lambda_{i}=\beta \times \overset{6}{\underset{j=1}{\sum }}\frac{C_{ij}\times \left(I_{j}+IV_{j}\right)}{N}$. The model parameters in Formula (1) are shown in Table 1, where *N* is the sum of the total population of each compartment; β is the potential of an individual being infected by contact once with an infectious person; and ε is reduced susceptibility. Note that *C*_*ij*_ represents an element in the contact matrix, reflecting the level of contact in the South African population, which is a 6 × 6 matrix (the detailed estimation of the contact matrix is shown in Supplementary Section 1). We assume that vaccines are rolled out *M*_1_ and *M*_2_ and doses are available each day, which are used for first and second injections, respectively. Vaccinated individuals may not be protected from infection due to immunity waning, we assume that individuals who received one and two doses lose vaccine protection with probabilities of η_1_ and η_2_, respectively. In addition, considering that the risk of severe disease of infection with Omicron is lower than that of Delta virus, the risk of hospitalization is also reduced; and patients who have been vaccinated and infected with the omicron variant can be cured through non-hospital treatment, such as the use of drugs and home isolation (21). We also assume that unvaccinated subjects moved to the recovered (*R*) compartment after they received intensive care (*Q*) or non-hospitalized treatment with probabilitiesμ and σ_2_; vaccinated subjects are assumed to have no risk of intensive care and just recover at a given rate σ_1_ (28).

**Table 1:** Descriptions of parameters.

**Variables**	**Description**	**Initial value**	**Resource**
*S*_*i*_	Susceptible population of age group *i*	22,563,300; 10,695,600; 8,949,400; 5,054,400; 4,861,900; 4,594,700	(21)
*V*1_*i*_	Vaccinated first dose population of age group *i*	10,000; 90,000; 80,000; 80,000; 30,000; 10,000	Assumed
V2_i_	Vaccinated second dose population of age group *i*	20,000; 600,000; 340,000; 270,000; 120,000; 80,000	(22)
*p*1_*i*_	Proportion of vaccinated first dose of age group *i*	-	Estimated
*p*2_*i*_	Proportion of vaccinated second dose of age group *i*	-	Estimated
M_1_	Vaccinated first dose population daily	-	Estimated
M_2_	Vaccinated second dose population daily	-	Estimated
*N*	The total contact possible population	59,300,000	(22)
**Parameters**	**Description**	**Value**	**Resource**
*C*_*ij*_	Number of contacts made by a person in age group *j* with people in age group *i*	Appendix	(23)
β	Probability of infected individuals transmission per contact	0.1	(24)
1/γ	Latent period without vaccination	5	(25)
μ	Recovery rate	0.25	(25)
δ	Nucleic acid test done per day	100,000	(20)
1/σ_1_	Self-recovery period after vaccination	21	(10)
1/σ_2_	Self-recovery period without vaccination	21	Assumed
η_1_	Probability of daily immune escape in individuals vaccinated first dose	0.129	(26)
η_2_	Probability of daily immune escape in individuals vaccinated second dose	0.093	(26)
ε	Reduced susceptibility	0.8	(27)
1/θ	Latent period after vaccination	5	Assumed
*d*_*i*_	Case fatality rate	0.00002; 0.000339; 0.000339; 0.000339; 0.00252; 0.00644	(25)

*In each cell for the fitted S_i_, E_i_, I_i_, d_i_ and so on, the values from left to right are for the age groups of 0–20, 21–30, 31–40, 41–50, 51–60, and 60+, respectively*.

In this study, the effective reproduction number that characterizes the mean number of secondary cases infected by a single infectious individual is calculated as *R*_*t*_ = ρ(*G*), where ρ is the spectral radius of the next generation matrix *G*. *F*(*x*) and *V*(*x*) are derived as follows:


(2) $$\begin{array}{c} F(x)=\left(\begin{array}{c} S_{i}\;*\;\lambda_{i}\\ 0\\ SV_{i}\;*\;\varepsilon \;*\;\lambda_{i}\\ 0 \end{array}\right)\lambda_{i}=\beta \times {\sum^{\text{​}}}_{j=1}^{6}\frac{C_{ij}\times (I_{j}+IV_{j})}{N} \end{array}$$

(3) $$\begin{array}{c} V(x)=\left(\begin{array}{c} E_{i}\;*\;\gamma \\ -E_{i}\;*\;\gamma +I_{i}\;*\;\frac{\delta }{N}+I_{i}\;*\;\sigma_{2}\\ EV_{i}\;*\;\theta \\ -EV_{i}\;*\;\theta +IV_{i}\;*\;\sigma_{1} \end{array}\right) \end{array}$$

Hence, one can obtain the next generation matrix *G* as:*G* = *FV*^−1^. Supplementary Section 2 presents the detailed derivation of the above equation for *R*_*t*_.

### Vaccination Strategies

In our study, we separated the population into six age groups 0–20, 21–30, 31–40, 41–50, 51–60 and older (> 60 years) by 50% of the amount of vaccine followed by a priority strategy of distributing vaccination proportionally to the population of other age groups under vary case. We referred to the strategies for prioritizing vaccinations for the 0–20, 21–30, 31–40, 41–50, 51–60, and 60+ years age groups as “0–20 first” and “21–30 first.” “31–40 first,” “41–50 first,” “51–60 first,” and “60+ first,” respectively. We simulated vaccine strategies under different vaccine supply plans, testing volumes, dose availability, infection rate, and other control measures (i.e., no control measures, moderate control measures, and strong control measures. In the absence of controls, the four positions are equally weighted. Under the strong control measures, the weights of the “at home,” “at work,” “at school,” and “other” matrices are 0.5, 1.2, 0, and 0.8, respectively, estimated from Google's mobile data during the lockdown (1 November 2021–31 January 2022). The weights under the moderate control measure are simulated by the average between the no control and strong control measure weights. Meanwhile, the effective reproduction number, cumulative infections, and deaths are the main indicators of infectious disease severity and public health problems, and can thus use to assess the effectiveness of different vaccination strategies.

## Results

**Figure 2:**
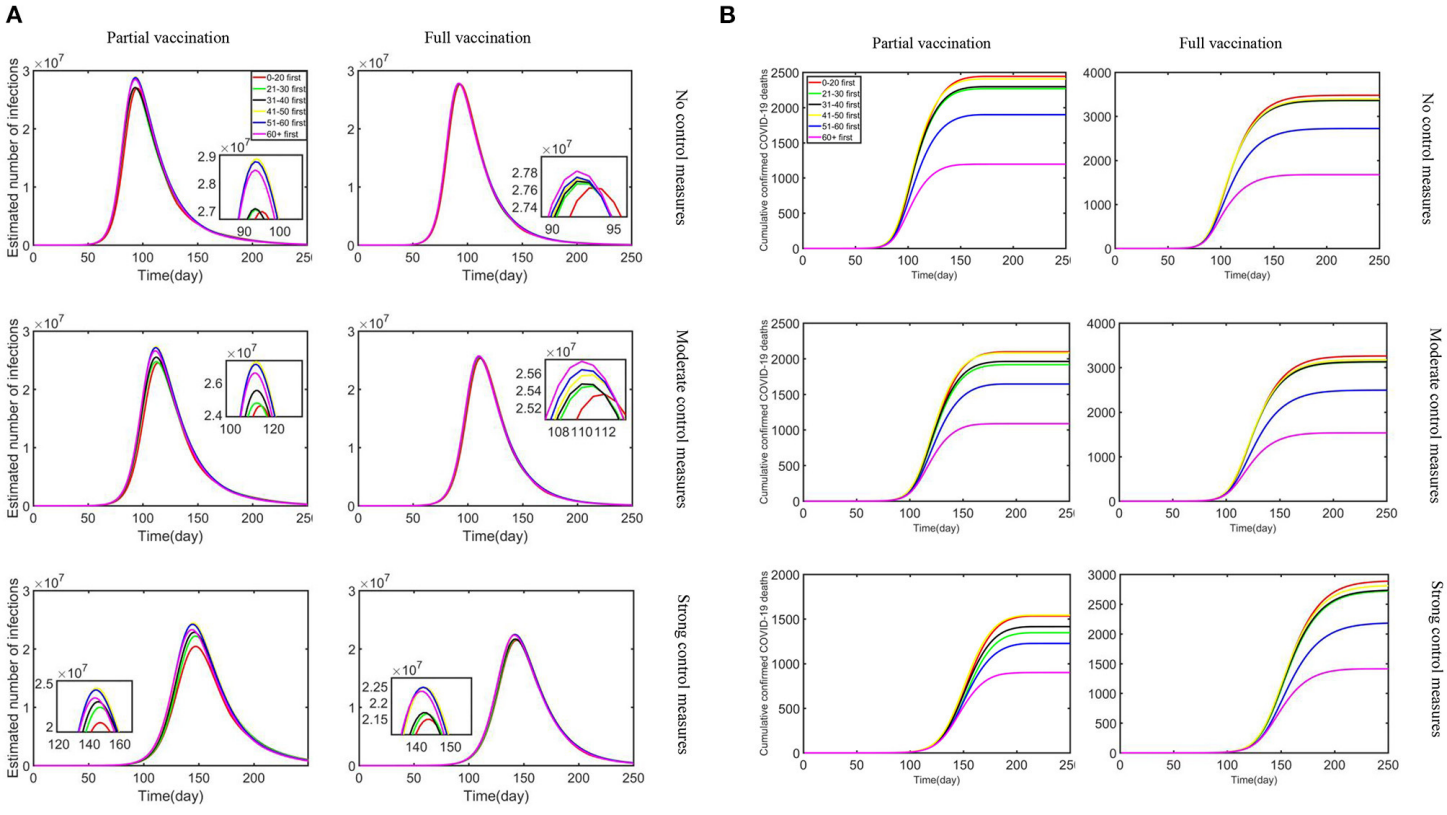
The combined impact of different vaccine supply plans and control measures on reductions in the estimated number of infections and cumulative confirmed deaths: **(A)** estimated number of infections; **(B)** cumulative confirmed COVID-19 deaths.

First, since only about 1% of South Africans are currently vaccinated for the third dose, we considered two overall vaccination effects with all for the first dose (partial vaccination) and all for the second doses (full vaccination) for the sensitivity analysis and compared the influence vaccination priority strategies exerted on the estimated number of confirmed cases and cumulative confirmed deaths under different control measures and vaccine supply plans, as shown in Figure 2.

With the strengthening of control measures, the peak number of daily infections is reduced, and the outbreak time is relatively delayed. Our research showed that the outbreak of Omicron is basically under control in about 200 days, which is in line with Nicole Wolter's research published in the Lancet (29). Moreover, the “0–20 first” strategy turned out to be the most effective in terms of any vaccine supply plans and control measures to reduce infections. In addition, “21–30 first” and “31–40 first” strategies have also had a positive effect. In particular, the reduction rate difference is minimal under no control measures. Partial vaccination that confers sterilizing immunity appears to minimize the extent of infection waves compared with full vaccination. Meanwhile, the most effective strategy to reduce cumulative confirmed deaths was the “60+ first” strategy under strong control measures and partial vaccination. The “51–60 first” strategy also produced relatively benefits although not optimal, and other strategies resulted in relatively similar reductions in deaths. As the control measures tightened under the partial vaccination, the effect of reducing the number of deaths became more pronounced. Obviously, under the same control measures, partial vaccination was more effective in reducing cumulative confirmed deaths than full vaccination. In the context of a vaccine shortage in South Africa, partial vaccination resulted in lower numbers of infections and deaths under different control measures compared with full vaccination. All measures led to a reduction in deaths and infections, but the “60+ first” strategy exerted obvious benefits compared with other measures. Meanwhile, reduction in infections is substantially effective with the strategy “0–20 first.”

Second, we investigated the effect of doses available each day on reducing the estimated number of infections and cumulative confirmed deaths under the combined effect of varying daily testing volume and infection rate. To research the fastest way to control the epidemic, we just studied the scenario with strong control measures and partial vaccination, which resulted in the maximum reduction in infections and deaths.

**Figure 3:**
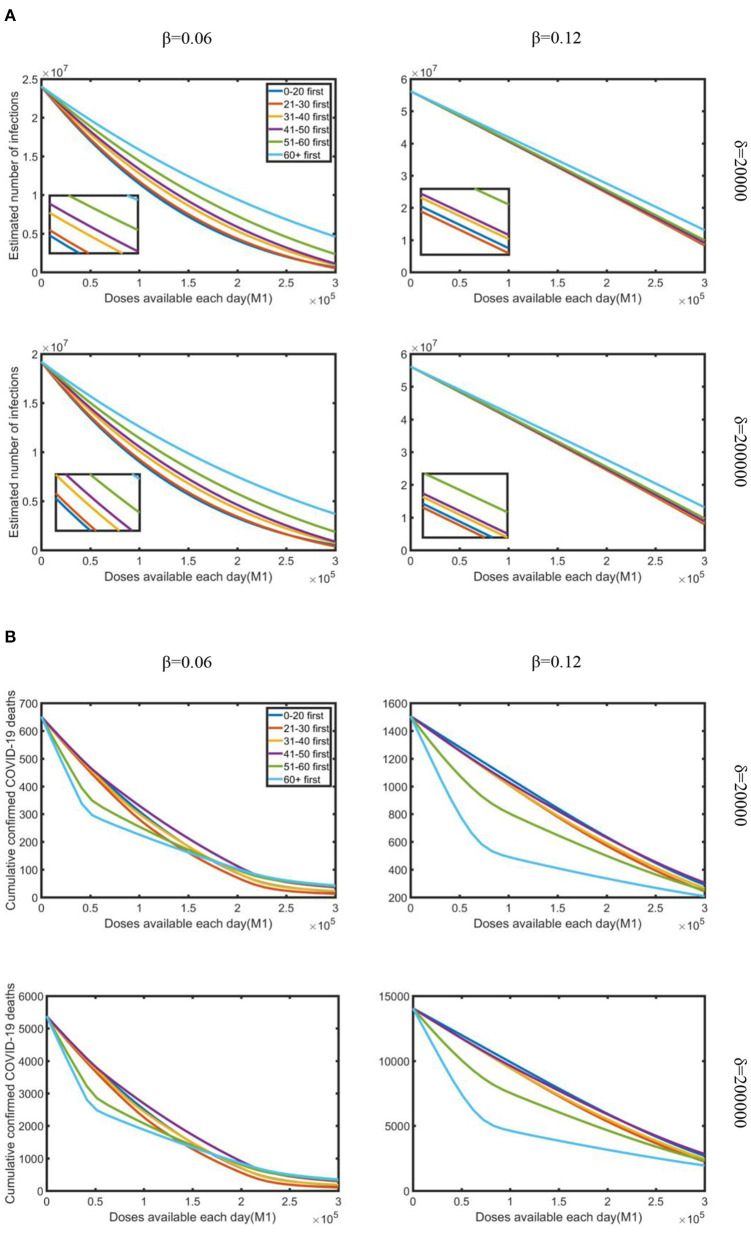
Under strong control measures, the impact of doses available each day on the reduction in the estimated number of infections and cumulative deaths occurred for various daily testing volumes (δ) and infection rate (β): **(A)** estimated a number of infections; **(B)** cumulative confirmed COVID-19 deaths. Testing volume is 20,000 and 200,000, which represent the minimum and maximum testing volume in South Africa during the research period.

As shown in Figure 3, as doses available each day increased, the estimated number of infections and cumulative deaths decreased regardless of daily testing volume or infection rate. When the infection rate was relatively lower, prioritizing vaccine allocation among the 0–20 age group consistently resulted in the greatest reduction in infections, but with a higher infection rate, the “21–30 first” strategy was the best. When doses available each day were relatively limited, the difference in reduction rate of the six strategies was minimal. However, when doses available each day were increased, the “0–20 first” and “21–30 first” strategies accompanied by apparent differences compared with other age groups. On the other hand, under varying doses availability, testing volume, and infection rate, all vaccination strategies produced a significant reduction in death. Under lower infection rate, when doses available each day were relatively limited, the “60+ first” strategy was the most effective strategy in reducing cumulative confirmed deaths, but as supply increased, “51–60 first” was the best strategy. While under a higher infection rate, the “60+ first” strategy resulted in a relatively more obvious effect than the other strategies. We also observed that the number of confirmed deaths in the lack of daily testing volume is less than that in the plenty of testing volume, while as the amount of testing increased, the number of deaths increased on the contrary.

Third, considering “0–20 first” and “60+ first” were the most effective strategies to reduce infections and deaths under strong control measures. We studied the impact of the varying proportion of vaccination priority under varying strategies to minimize the cumulative morbidities and mortalities. After the target age group has been vaccinated the assumed vaccine proportion, vaccines are distributed to the remaining groups proportionally to the size of the remaining age groups. We used different priority vaccination rates in the simulations and assumed a fixed daily dose was available.

**Figure 4:**
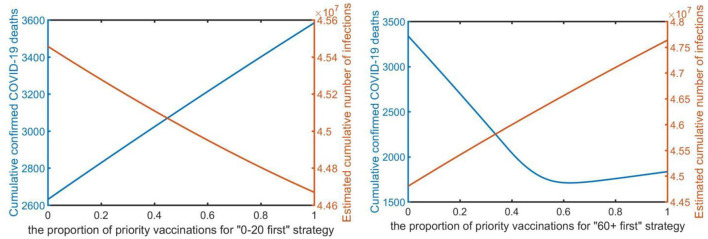
The proportion of the vaccination priority under the “0–20 first” strategy and “60+ first” strategy (*x*-axis) to minimize the total number of infections and deaths (*y*-axis).

We assumed that, among different vaccination priority ratios, vaccines are initially vaccinated to the target age group by x% of the vaccine quantity, and then to other age groups in proportion to their population, where x ranged from 0 to 100%. Simulations were performed using a daily testing volume of sixty thousand under strong control (i.e., the infection rate is 0.1) with partial vaccination. From Figure 4, under the “0–20 first” strategy, as a proportion of priority vaccinations increased, the number of confirmed deaths increased by a certain margin because of the epidemic spread, but the number of confirmed cases decreased, and always had a favorable effect on reducing infections. As presented in the Supplemental Section 3, under the “21–30 first” and “31–40 first” strategies, increasing the proportion of priority vaccinations has a similar effect, but the “0–20 first” strategy has a more pronounced effect. However, under the “60+ first” strategy, as a proportion of priority vaccinations increased, the number of confirmed infections increased, which resulted in a noticeable impact on the decline in the number of confirmed deaths. In particular, when the proportion of priority vaccinations reached 60%, the “60+ first” strategy led to the greatest reduction in deaths. However, after reaching the 60% proportion in the vaccination priority strategy, the number of cumulative confirmed deaths slightly increased. Figure 4 illustrates that prioritizing vaccine allocation among the 60+ age group with 60% of the total amount of vaccine consistently resulted in the greatest reduction in deaths. Overall reduction in infections is strongly limited by the vaccine proportion of the target age group, with 60% vaccine proportion leading to the most reduced mortality rate.

Lastly, we conducted a sensitivity analysis concerning the effect of each priority strategy on the effective reproduction number (*R*_*t*_).

**Figure 5:**
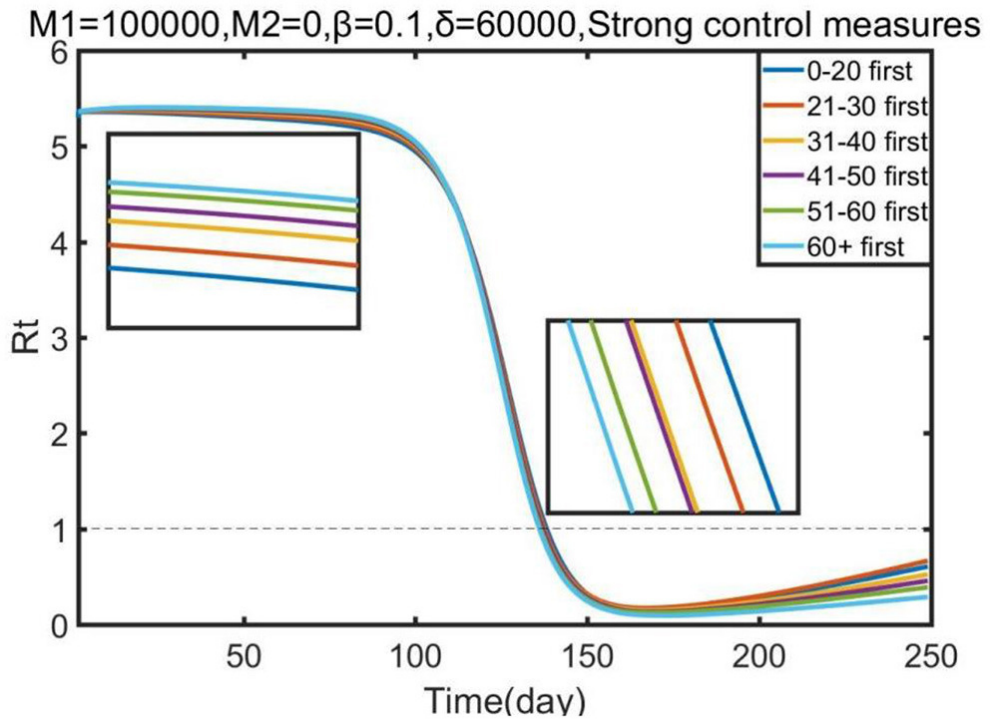
Effect of each priority vaccination strategy on the reduction in the effective reproduction number (*R*_*t*_).

Figure 5 illustrates that all vaccination strategies were the best strategies to reduce the effective reproduction number, although all strategies resulted in similar reductions. However, in the early stage of Omicron, prioritizing vaccine allocation for the 0–20 age group resulted in the greatest reduction in effective reproduction number compared with other strategies; but in the later stage, more vaccines should be allocated to other age groups, such as the 60+ age group and the 51–60 age group. Our research showed that the effective reproduction number (*R*_*t*_) was reduced to 1 at ~150 days, which means that the epidemic would be controlled. Thus, we suggest that the government should prioritize vaccine allocation for the 0–20 age group, and then guarantee the vaccination of other age groups, to control the epidemic as soon as possible.

## Discussions

Study on Omicron shows that it is high infectivity and has a greater ability to evade immunity than Delta (30). Moreover, Omicron appears to cause less severe infections and a higher chance of reinfection compared to previous variants (31). The reduction in hospital admissions and severity may be caused by previous high levels of infection, improved vaccination coverage and reduced pathogenicity or virulence of Omicron variant (32). Despite these, vaccination is still an important measure in protecting the population. This research lead to the adoption of an age-stratified modeling method to assess and compare vaccine prioritization strategies for COVID-19. The total population is separated into six age groups and the impact of prioritizing vaccination for target age groups on reducing the number of confirmed cases and deaths were compared under various potential vaccine characteristics and hypothetical scenarios, such as the control measures, vaccine availability, testing rate, and rollout speed of vaccine. It is worth noting that the choice of age group width will affect our results. Based on the assumption that the age distribution of morbidity and mortality are smooth, we divided South Africa's population into 10-year age groups. If ages were grouped too widely, it might hide actual age-specific case-fatality, social contacts and contact patterns differences (33).

Our results indicated that in light of South Africa's demographics, vaccinating older age groups (>60 years) largely reduced the cumulative deaths in all scenarios considered, and was in line with prior work also (13, 34). By contrast, prioritizing 0–20 age group who usually possess a higher contact rate exerts a greater effect on reducing morbidities relative to prioritizing older age groups. Furthermore, compared to the third wave, the data from Daily Hospital Surveillance (DATCOV) report showed a higher proportion of hospital admissions for patients under 20 in the early fourth wave (35). This is likely attributable to the fact that in the previous waves of the epidemic, the vaccination was mainly aimed at adults, and the distribution of vaccines to the adolescents was not advocated, which was mainly due to the lack of sufficient clinical data. Therefore, we recommend that the vaccine allocation strategy for the 0–20 age group should be refined based on actual clinical manifestations and characteristics. Some studies have advocated that the distribution of vaccines among young people should vaccinate 16-17 age group first, followed by 12–15 age group, and so on. However, there is no definite vaccination schedule with fixed age groups for young people (36). Our study didn't make too much subdivision discussion considering that adopting more complex division may incur unidentifiable issues caused by inadequate data in many involved compartments (37). Moreover, it is uncertain as to how susceptibility to children's infection changes with age (6).

Our analyses indicated that the combined effect of control measures and vaccine supply plan was the most effective way to reduce cumulative confirmed cases. We found that one dose of vaccine is more effective in minimizing severe COVID-19, which does not represent that the effect of vaccination of one dose is better than two-dose, while for the country with limited vaccine supply and low vaccination coverage, ensuring the first dose of vaccine supply will have a significant impact on severe disease. Correspondingly, in developed or developing countries where vaccine supply is unlimited, two-dose vaccines or even booster shots are significant to reduce disease (18).

We observed that, given the daily testing volume and infection rate, our model identified a few scenarios wherein prioritizing younger adults aged 0–20 and 60+ years would provide greater morbidity and mortality benefits, respectively. These scenarios were restricted to the conditions of inadequate vaccine supply and lower infection rates. We also found that the number of confirmed deaths in the lack of daily testing volume is less than that in the plenty of testing volume, while as the amount of testing increased, the number of deaths increased on the contrary. This could be explained by the fact that increasing the number of daily tests could identify more infected people, conversely, relatively inadequate testing rates will lead to more undetected infections among the susceptible, which may mislead our control measures of COVID-19. Thus, the symptomatic and asymptomatic patients must be tested to identify infectious individuals, and take clinical treatment to prevent the onward infection of others, which results in reducing the number of infected and deaths.

Besides, modeling for COVID-19 vaccination has discovered that the optimal balance between vaccine allocation and a total number of deaths depends on the proportion of priority vaccination, recommending the vaccination of the 60+ age group for 60% vaccine proportion. However, this recommendation is sensitive to the proportion of priority vaccination because, when the proportion of priority vaccination exceeds 60%, the effect shifts toward the opposite. These results can be illustrated by the features of the population ratio. If we continue to increase the proportion of priority vaccination to 60+ age group, it will reduce the proportion of other people who have the higher population ratio and higher contact rate, resulting in the increasing mortality in other age group. We also observed that the increase in the proportion of vaccines given priority to 0–20 groups always had a favorable effect on reducing infections. We then examined the effects of each priority strategy on the reduction of the effective reproduction number (*R*_*t*_). We observed that significant distinction among COVID-19 vaccine allocation policies for relative reductions in *R*_*t*_. The results suggest that the public health authorities should give priority to supplying the 0–20 age group, and then allocating vaccines for remaining age groups. The speed of COVID-19 vaccination is pivotal to rapid epidemic containment, however, vaccine hesitancy is a major barrier to speed up inoculation and improve vaccination coverage (38, 39). Therefore, the government needs to provide sustained health education and communication to strengthen individual vaccine willingness (40, 41).

Furthermore, our model can be modified to quantify and evaluate the effect of COVID-19 vaccine prioritization policies on cumulative incidence and mortality, facing the changes of the epidemic and s multitude of sequential waves in the case of COVID-19. Our study relies on actual epidemiological data and estimation of the related parameters (such as, infection force for each age group) and depends on pandemic contact patterns to the extent individuals interact with each other. Thus, within this framework, the model can incorporate epidemiological data in target areas and estimates of age-stratified contact rates to model future pandemic scenarios (42), and optimize the process for the evaluation of vaccine prioritization strategies against COVID-19. In particular, virus mutations characterized by increased contagiosity and relative capacity for immunological escape may trigger the decrease in vaccine effectiveness. The proposed model could provide an evidence-based rationale for prioritizing first-dose coverage and vaccination priorities based on the varying contributions of the vaccine effects. For example, in countries where vaccine coverage is constrained by supply, high first-dose coverage is important to minimize severe disease. Meanwhile, in high-income and high-middle-income countries, attention has turned to breakthrough infections and waning immunity (43).

Besides, our framework can be adapted to consider more possible goals of vaccination, such as minimizing hospitalizations, comorbidities, or economic costs (44) based on the development of future pandemic scenarios. Our studies could help evaluate control measures' performance and improve vaccine allocation strategy for COVID-19 epidemic.

## Data Availability Statement

The original contributions presented in the study are included in the article/Supplementary Material, further inquiries can be directed to the corresponding authors.

## Author Contributions

CZ and YL proposed a framework and implemented the simulation experiments. ZM, FZ, and YZ contributed to model building, data analysis, and writing the article. All authors contributed to the article and approved the submitted version.

## Funding

This work was supported by the National Social Science Foundation of China (Grant No. 21BGL298) and the Fundamental Research Funds for the Provincial Universities of Zhejiang (Grant No. JR202203).

## Conflict of Interest

The authors declare that the research was conducted in the absence of any commercial or financial relationships that could be construed as a potential conflict of interest.

## Publisher's Note

All claims expressed in this article are solely those of the authors and do not necessarily represent those of their affiliated organizations, or those of the publisher, the editors and the reviewers. Any product that may be evaluated in this article, or claim that may be made by its manufacturer, is not guaranteed or endorsed by the publisher.
