# Supplementary material for: Assessing Vaccination Prioritization Strategies for COVID-19 in South Africa Based on Age-Specific Compartment Model
Source: Front Public Health. 2022 Jun 15;10:876551. doi: 10.3389/fpubh.2022.876551 (PMC9240634; doi:10.3389/fpubh.2022.876551)
Supplement: Supplementary file 1 [file Data_Sheet_1.pdf]

SUPPLEMENTAL FILE

Contents

1. Contact matrices 2
2. Estimated the effective reproduction number R_t 2
3.Simulations with varying priority group 4

1. Contact matrices

C_{ij} is an entry of the contact matrix, which reflecting the level of contact between different age groups. We parameterized C_{ij} using specific Contact matrices (C_M) that were construct based on social mixing patterns in South Africa population to match age group we defined. The contact matrix of South Africa（C_M）which is 16 × 16 where each element m_{ij} is the mean number of contacts an individual in age group i makes with individuals in age group j per day. u_i is the population of age group i, which is gained from statistics website (www.PopulationPyramid.net), T_{ij} is the total number of contacts between age groups i and j; $T_{ij}=m_{ij}*u_i$.

The detailed computation for contact matrix as follows:

Diagonal entries of C_{ij} ($i = j$) are:

$$Cij = \frac{T_{2i-12i-1} + \frac{1}{2}(T_{2i-12i} + T_{2i2i-1}) + T_{2i2i}}{u_{2i-1} + u_{2i}}$$

Non-diagonal entries of C_{ij} are:

$$Cij = \frac{(T_{2i-12j-1} + T_{2j-12i-1}) + (T_{2i-12j} + T_{2j2i-1}) + (T_{2i2j-1} + T_{2j-12i}) + (T_{2i2j} + T_{2j2i})}{2(u_{2i-1} + u_{2i})}$$

We present the graph of the portion of individuals from South Africa population in each age group and contact matrices for each of four locations and three control measures.

Table S.1: Contact matrix for South Africa

home	0−20	20−30	30−40	40−50	50−60	60+
0−20	1.478	0.593	0.912	0.652	0.317	0.314
20−30	1.177	0.998	0.277	0.26	0.306	0.101
30−40	2.2	0.337	0.581	0.185	0.133	0.16
40−50	2.3	0.462	0.27	0.376	0.164	0.157
50−60	1.453	0.709	0.254	0.213	0.292	0.205
60+	1.409	0.243	0.317	0.213	0.213	0.197

school	0−20	20−30	30−40	40−50	50−60	60+
0−20	4.754	0.164	0.246	0.155	0.135	0.017
20−30	0.325	0.408	0.093	0.045	0.02	0.004
30−40	0.592	0.113	0.112	0.074	0.028	0.009
40−50	0.546	0.08	0.109	0.065	0.05	0.009
50−60	0.62	0.045	0.053	0.067	0.037	0.013
60+	0.076	0.01	0.017	0.0117	0.013	0.02

work	0−20	20−30	30−40	40−50	50−60	60+
0−20	0.285	0.265	0.186	0.157	0.092	0.024
20−30	0.525	1.503	1.419	0.971	0.583	0.147
30−40	0.448	1.724	1.684	1.614	0.91	0.19
40−50	0.555	1.726	2.361	1.618	1.359	0.269
50−60	0.422	1.348	1.732	1.768	1.103	0.308
60+	0.109	0.353	0.375	0.363	0.32	0.073

other	0−20	20−30	30−40	40−50	50−60	60+
0−20	5.062	1.267	0.705	0.375	0.177	0.155
20−30	2.514	4.127	1.303	0.493	0.303	0.108
30−40	1.7	1.583	1.163	0.549	0.293	0.155
40−50	1.323	0.876	0.804	0.4006	0.238	0.127
50−60	0.811	0.701	0.557	0.31	0.208	0.142
60+	0.694	0.259	0.306	0.171	0.147	0.11

No control measures	0−20	20−30	30−40	40−50	50−60	60+
0−20	11.58	2.288	2.048	1.339	0.721	0.509
20−30	4.541	7.036	3.092	1.769	1.212	0.36
30−40	4.939	3.757	3.54	2.422	1.364	0.514
40−50	4.724	3.145	3.544	2.46	1.813	0.561
50−60	3.306	2.803	2.596	2.359	1.64	0.668
60+	2.288	0.865	1.015	0.758	0.693	0.4

Moderate control measures	0−20	20−30	30−40	40−50	50−60	60+
0−20	8.355	1.958	1.646	1.077	0.565	0.41
20−30	3.885	6.32	2.99	1.729	1.153	0.337
30−40	3.968	3.63	3.391	2.445	1.378	0.473
40−50	3.799	3.075	3.578	2.455	1.858	0.532
50−60	2.593	2.668	2.623	2.418	1.638	0.627
60+	1.839	0.808	0.934	0.718	0.65	0.337

Strong control measures	0−20	20−30	30−40	40−50	50−60	60+
0−20	5.131	1.628	1.243	0.815	0.41	0.31
20−30	3.23	5.604	2.883	1.69	1.095	0.313
30−40	2.997	3.504	3.241	2.469	1.393	0.432
40−50	2.874	3.004	3.611	2.45	1.903	0.502
50−60	1.881	2.533	2.651	2.477	1.636	0.586
60+	1.391	0.752	0.853	0.678	0.608	0.274

2. Estimated the effective reproduction number R_t

In this study, the effective reproduction number that characterizes the mean number of secondary cases infected by a single infectious individual is calculated as $R_t = \rho(G)$, where ρ is the spectral radius of the next generation matrix G. $F(x)$ and $V(x)$ are derived as follows:

$$F(x) = \begin{pmatrix} S_i * \lambda_i \\ 0 \\ SV_i * \varepsilon * \lambda_i \\ 0 \end{pmatrix} \quad \lambda_i = \beta \times \sum_{j=1}^{6} \frac{C_{ij} \times (I_j + IV_j)}{N}$$

$$V(x) = \begin{pmatrix} E_i * \gamma \\ -E_i * \gamma + I_i * \frac{\delta}{N} + I_i * \sigma_2 \\ EV_i * \theta \\ -EV_i * \theta + IV_i * \sigma_1 \end{pmatrix}$$

Thus, F and V are $24 * 24$ matrices at x_0 given by

$$\mathrm{F}=\begin{pmatrix} 0 & F_{12} & 0 & F_{14} \\ 0 & 0 & 0 & 0 \\ 0 & F_{32} & 0 & F_{34} \\ 0 & 0 & 0 & 0 \end{pmatrix}$$

$$F_{12}=F_{14}=\begin{pmatrix} \beta * c_{11} * \frac{S_1}{N} & \beta * c_{12} * \frac{S_1}{N} & \beta * c_{13} * \frac{S_1}{N} & \beta * c_{14} * \frac{S_1}{N} & \beta * c_{15} * \frac{S_1}{N} & \beta * c_{16} * \frac{S_1}{N} \\ \beta * c_{21} * \frac{S_2}{N} & \beta * c_{22} * \frac{S_2}{N} & \beta * c_{23} * \frac{S_2}{N} & \beta * c_{24} * \frac{S_2}{N} & \beta * c_{25} * \frac{S_2}{N} & \beta * c_{26} * \frac{S_2}{N} \\ \beta * c_{31} * \frac{S_3}{N} & \beta * c_{32} * \frac{S_3}{N} & \beta * c_{33} * \frac{S_3}{N} & \beta * c_{34} * \frac{S_3}{N} & \beta * c_{35} * \frac{S_3}{N} & \beta * c_{36} * \frac{S_3}{N} \\ \beta * c_{41} * \frac{S_4}{N} & \beta * c_{42} * \frac{S_4}{N} & \beta * c_{43} * \frac{S_4}{N} & \beta * c_{44} * \frac{S_4}{N} & \beta * c_{45} * \frac{S_4}{N} & \beta * c_{46} * \frac{S_4}{N} \\ \beta * c_{51} * \frac{S_5}{N} & \beta * c_{52} * \frac{S_5}{N} & \beta * c_{53} * \frac{S_5}{N} & \beta * c_{54} * \frac{S_5}{N} & \beta * c_{55} * \frac{S_5}{N} & \beta * c_{56} * \frac{S_5}{N} \\ \beta * c_{61} * \frac{S_6}{N} & \beta * c_{62} * \frac{S_6}{N} & \beta * c_{63} * \frac{S_6}{N} & \beta * c_{64} * \frac{S_6}{N} & \beta * c_{65} * \frac{S_6}{N} & \beta * c_{66} * \frac{S_6}{N} \end{pmatrix}$$

$$F_{32}=F_{34}=\begin{pmatrix} \beta * \varepsilon * c_{11} * \frac{SV_1}{N} & \beta * \varepsilon * c_{12} * \frac{SV_1}{N} & \beta * \varepsilon * c_{13} * \frac{SV_1}{N} & \beta * \varepsilon * c_{14} * \frac{SV_1}{N} & \beta * \varepsilon * c_{15} * \frac{SV_1}{N} & \beta * \varepsilon * c_{16} * \frac{SV_1}{N} \\ \beta * \varepsilon * c_{21} * \frac{SV_2}{N} & \beta * \varepsilon * c_{22} * \frac{SV_2}{N} & \beta * \varepsilon * c_{23} * \frac{SV_2}{N} & \beta * \varepsilon * c_{24} * \frac{SV_2}{N} & \beta * \varepsilon * c_{25} * \frac{SV_2}{N} & \beta * \varepsilon * c_{26} * \frac{SV_2}{N} \\ \beta * \varepsilon * c_{31} * \frac{SV_3}{N} & \beta * \varepsilon * c_{32} * \frac{SV_3}{N} & \beta * \varepsilon * c_{33} * \frac{SV_3}{N} & \beta * \varepsilon * c_{34} * \frac{SV_3}{N} & \beta * \varepsilon * c_{35} * \frac{SV_3}{N} & \beta * \varepsilon * c_{36} * \frac{SV_3}{N} \\ \beta * \varepsilon * c_{41} * \frac{SV_4}{N} & \beta * \varepsilon * c_{42} * \frac{SV_4}{N} & \beta * \varepsilon * c_{43} * \frac{SV_4}{N} & \beta * \varepsilon * c_{44} * \frac{SV_4}{N} & \beta * \varepsilon * c_{45} * \frac{SV_4}{N} & \beta * \varepsilon * c_{46} * \frac{SV_4}{N} \\ \beta * \varepsilon * c_{51} * \frac{SV_5}{N} & \beta * \varepsilon * c_{52} * \frac{SV_5}{N} & \beta * \varepsilon * c_{53} * \frac{SV_5}{N} & \beta * \varepsilon * c_{54} * \frac{SV_5}{N} & \beta * \varepsilon * c_{55} * \frac{SV_5}{N} & \beta * \varepsilon * c_{56} * \frac{SV_5}{N} \\ \beta * \varepsilon * c_{61} * \frac{SV_6}{N} & \beta * \varepsilon * c_{62} * \frac{SV_6}{N} & \beta * \varepsilon * c_{63} * \frac{SV_6}{N} & \beta * \varepsilon * c_{64} * \frac{SV_6}{N} & \beta * \varepsilon * c_{65} * \frac{SV_6}{N} & \beta * \varepsilon * c_{66} * \frac{SV_6}{N} \end{pmatrix}$$

$$\mathrm{V}=\begin{pmatrix} V_{11} & 0 & 0 & 0 \\ V_{21} & V_{22} & 0 & 0 \\ 0 & 0 & V_{33} & 0 \\ 0 & 0 & V_{43} & V_{44} \end{pmatrix}$$

$$V_{11}=\begin{pmatrix} \gamma & 0 & 0 & 0 & 0 & 0 \\ 0 & \gamma & 0 & 0 & 0 & 0 \\ 0 & 0 & \gamma & 0 & 0 & 0 \\ 0 & 0 & 0 & \gamma & 0 & 0 \\ 0 & 0 & 0 & 0 & \gamma & 0 \\ 0 & 0 & 0 & 0 & 0 & \gamma \end{pmatrix}, V_{21}=\begin{pmatrix} -\gamma & 0 & 0 & 0 & 0 & 0 \\ 0 & -\gamma & 0 & 0 & 0 & 0 \\ 0 & 0 & -\gamma & 0 & 0 & 0 \\ 0 & 0 & 0 & -\gamma & 0 & 0 \\ 0 & 0 & 0 & 0 & -\gamma & 0 \\ 0 & 0 & 0 & 0 & 0 & -\gamma \end{pmatrix}, V_{22}=\begin{pmatrix} \sigma_2+\frac{\delta}{N} & 0 & 0 & 0 & 0 & 0 \\ 0 & \sigma_2+\frac{\delta}{N} & 0 & 0 & 0 & 0 \\ 0 & 0 & \sigma_2+\frac{\delta}{N} & 0 & 0 & 0 \\ 0 & 0 & 0 & \sigma_2+\frac{\delta}{N} & 0 & 0 \\ 0 & 0 & 0 & 0 & \sigma_2+\frac{\delta}{N} & 0 \\ 0 & 0 & 0 & 0 & 0 & \sigma_2+\frac{\delta}{N} \end{pmatrix},$$

$$V_{33}=\begin{pmatrix} \theta & 0 & 0 & 0 & 0 & 0 \\ 0 & \theta & 0 & 0 & 0 & 0 \\ 0 & 0 & \theta & 0 & 0 & 0 \\ 0 & 0 & 0 & \theta & 0 & 0 \\ 0 & 0 & 0 & 0 & \theta & 0 \\ 0 & 0 & 0 & 0 & 0 & \theta \end{pmatrix}, V_{43}=\begin{pmatrix} -\theta & 0 & 0 & 0 & 0 & 0 \\ 0 & -\theta & 0 & 0 & 0 & 0 \\ 0 & 0 & -\theta & 0 & 0 & 0 \\ 0 & 0 & 0 & -\theta & 0 & 0 \\ 0 & 0 & 0 & 0 & -\theta & 0 \\ 0 & 0 & 0 & 0 & 0 & -\theta \end{pmatrix}, V_{44}=\begin{pmatrix} \sigma_1 & 0 & 0 & 0 & 0 & 0 \\ 0 & \sigma_1 & 0 & 0 & 0 & 0 \\ 0 & 0 & \sigma_1 & 0 & 0 & 0 \\ 0 & 0 & 0 & \sigma_1 & 0 & 0 \\ 0 & 0 & 0 & 0 & \sigma_1 & 0 \\ 0 & 0 & 0 & 0 & 0 & \sigma_1 \end{pmatrix}$$

Hence, one can obtain the next generation matrix G as:

$$G=FV^{-1}=\begin{pmatrix} G_{11} & G_{12} \\ G_{21} & G_{22} \end{pmatrix}, G_{11}=G_{12}=\begin{bmatrix} \frac{\beta*C_{11}*S_1}{N*\sigma2+\delta} & \frac{\beta*C_{11}*S_1}{N*\sigma2+\delta} & \frac{\beta*C_{11}*S_1}{N*\sigma2+\delta} & \frac{\beta*C_{11}*S_1}{N*\sigma2+\delta} & \frac{\beta*C_{11}*S_1}{N*\sigma2+\delta} & \frac{\beta*C_{11}*S_1}{N*\sigma2+\delta} \\ \frac{\beta*C_{21}*S_2}{N*\sigma2+\delta} & \frac{\beta*C_{22}*S_2}{N*\sigma2+\delta} & \frac{\beta*C_{23}*S_2}{N*\sigma2+\delta} & \frac{\beta*C_{24}*S_2}{N*\sigma2+\delta} & \frac{\beta*C_{25}*S_2}{N*\sigma2+\delta} & \frac{\beta*C_{26}*S_2}{N*\sigma2+\delta} \\ \frac{\beta*C_{31}*S_3}{N*\sigma2+\delta} & \frac{\beta*C_{32}*S_3}{N*\sigma2+\delta} & \frac{\beta*C_{33}*S_3}{N*\sigma2+\delta} & \frac{\beta*C_{34}*S_3}{N*\sigma2+\delta} & \frac{\beta*C_{35}*S_3}{N*\sigma2+\delta} & \frac{\beta*C_{36}*S_3}{N*\sigma2+\delta} \\ \frac{\beta*C_{41}*S_4}{N*\sigma2+\delta} & \frac{\beta*C_{42}*S_4}{N*\sigma2+\delta} & \frac{\beta*C_{43}*S_4}{N*\sigma2+\delta} & \frac{\beta*C_{44}*S_4}{N*\sigma2+\delta} & \frac{\beta*C_{45}*S_4}{N*\sigma2+\delta} & \frac{\beta*C_{46}*S_4}{N*\sigma2+\delta} \\ \frac{\beta*C_{51}*S_5}{N*\sigma2+\delta} & \frac{\beta*C_{52}*S_5}{N*\sigma2+\delta} & \frac{\beta*C_{53}*S_5}{N*\sigma2+\delta} & \frac{\beta*C_{54}*S_5}{N*\sigma2+\delta} & \frac{\beta*C_{55}*S_5}{N*\sigma2+\delta} & \frac{\beta*C_{56}*S_5}{N*\sigma2+\delta} \\ \frac{\beta*C_{61}*S_6}{N*\sigma2+\delta} & \frac{\beta*C_{62}*S_6}{N*\sigma2+\delta} & \frac{\beta*C_{63}*S_6}{N*\sigma2+\delta} & \frac{\beta*C_{64}*S_6}{N*\sigma2+\delta} & \frac{\beta*C_{65}*S_6}{N*\sigma2+\delta} & \frac{\beta*C_{66}*S_6}{N*\sigma2+\delta} \end{bmatrix}$$

$$G_{21}=G_{22}=\begin{bmatrix} \frac{\varepsilon*\beta*C_{11}*SV_1}{N*\sigma2+\delta} & \frac{\varepsilon*\beta*C_{11}*SV_1}{N*\sigma2+\delta} & \frac{\varepsilon*\beta*C_{11}*SV_1}{N*\sigma2+\delta} & \frac{\varepsilon*\beta*C_{11}*SV_1}{N*\sigma2+\delta} & \frac{\varepsilon*\beta*C_{11}*SV_1}{N*\sigma2+\delta} & \frac{\varepsilon*\beta*C_{11}*SV_1}{N*\sigma2+\delta} \\ \frac{\varepsilon*\beta*C_{21}*SV_2}{N*\sigma2+\delta} & \frac{\varepsilon*\beta*C_{22}*SV_2}{N*\sigma2+\delta} & \frac{\varepsilon*\beta*C_{23}*SV_2}{N*\sigma2+\delta} & \frac{\varepsilon*\beta*C_{24}*SV_2}{N*\sigma2+\delta} & \frac{\varepsilon*\beta*C_{25}*SV_2}{N*\sigma2+\delta} & \frac{\varepsilon*\beta*C_{26}*S_{V2}}{N*\sigma2+\delta} \\ \frac{\varepsilon*\beta*C_{31}*SV_3}{N*\sigma2+\delta} & \frac{\varepsilon*\beta*C_{32}*SV_3}{N*\sigma2+\delta} & \frac{\varepsilon*\beta*C_{33}*SV_3}{N*\sigma2+\delta} & \frac{\varepsilon*\beta*C_{34}*SV_3}{N*\sigma2+\delta} & \frac{\varepsilon*\beta*C_{35}*SV_3}{N*\sigma2+\delta} & \frac{\varepsilon*\beta*C_{36}*SV_3}{N*\sigma2+\delta} \\ \frac{\varepsilon*\beta*C_{41}*SV_4}{N*\sigma2+\delta} & \frac{\varepsilon*\beta*C_{42}*SV_4}{N*\sigma2+\delta} & \frac{\varepsilon*\beta*C_{43}*SV_4}{N*\sigma2+\delta} & \frac{\varepsilon*\beta*C_{44}*SV_4}{N*\sigma2+\delta} & \frac{\varepsilon*\beta*C_{45}*SV_4}{N*\sigma2+\delta} & \frac{\varepsilon*\beta*C_{46}*SV_4}{N*\sigma2+\delta} \\ \frac{\varepsilon*\beta*C_{51}*SV_5}{N*\sigma2+\delta} & \frac{\varepsilon*\beta*C_{52}*SV_5}{N*\sigma2+\delta} & \frac{\varepsilon*\beta*C_{53}*SV_5}{N*\sigma2+\delta} & \frac{\varepsilon*\beta*C_{54}*SV_5}{N*\sigma2+\delta} & \frac{\varepsilon*\beta*C_{55}*SV_5}{N*\sigma2+\delta} & \frac{\varepsilon*\beta*C_{56}*SV_5}{N*\sigma2+\delta} \\ \frac{\varepsilon*\beta*C_{61}*SV_6}{N*\sigma2+\delta} & \frac{\varepsilon*\beta*C_{62}*SV_6}{N*\sigma2+\delta} & \frac{\varepsilon*\beta*C_{63}*SV_6}{N*\sigma2+\delta} & \frac{\varepsilon*\beta*C_{64}*SV_6}{N*\sigma2+\delta} & \frac{\varepsilon*\beta*C_{65}*SV_6}{N*\sigma2+\delta} & \frac{\varepsilon*\beta*C_{66}*SV_6}{N*\sigma2+\delta} \end{bmatrix}$$

Finally, the effective reproduction number R_t is computed as the spectral radius $\rho(G)$ of the next generate matrix G, i. e. $R_t=\rho(G)$.

3. Simulations with varying priority group

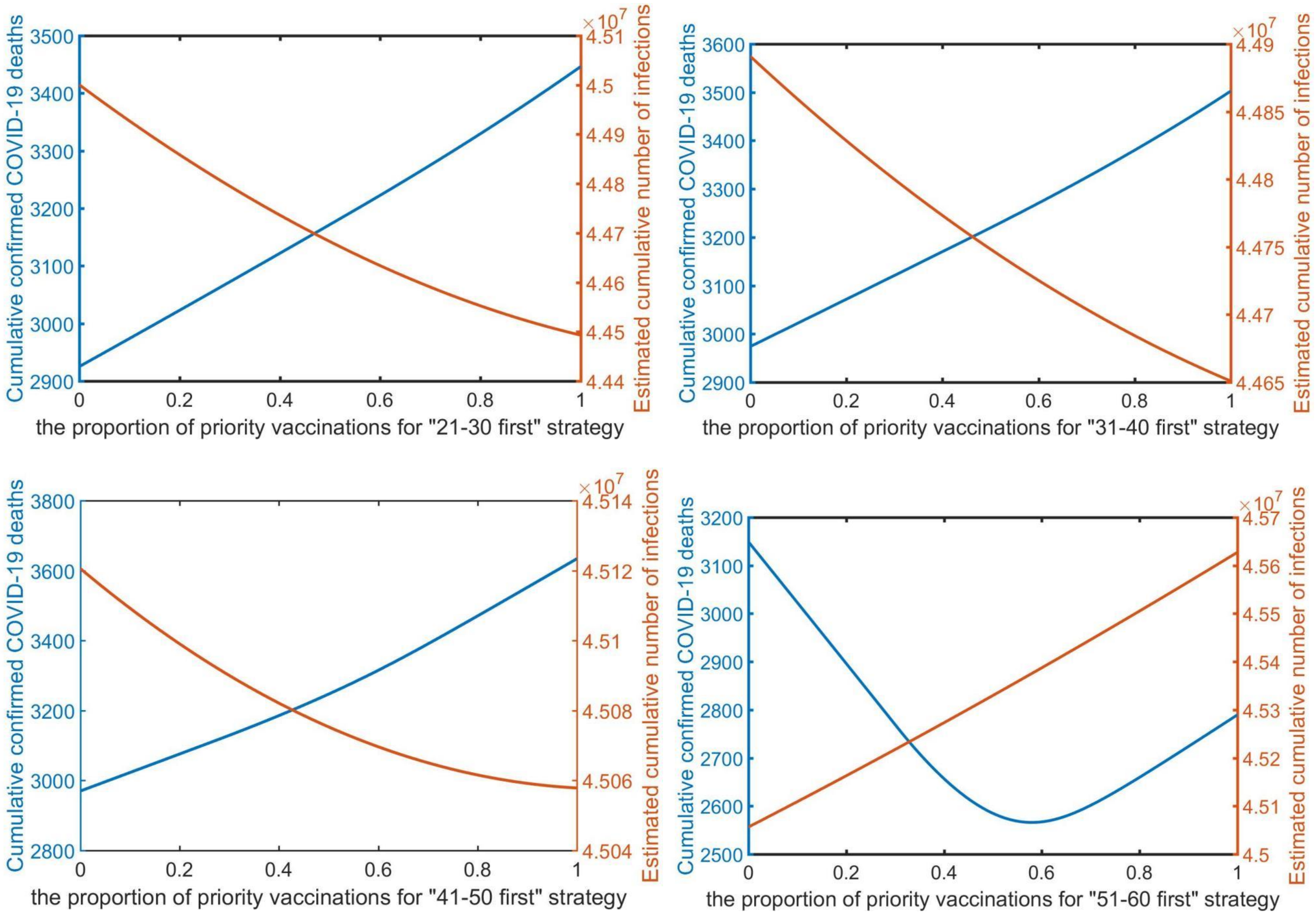

Figure S.1: Proportion of the vaccination priority under varying strategy (x-axis) to minimize the accumulative number of infections and deaths (y-axis).
